# An algorithm to extract three‐dimensional motion by marker tracking in the kV projections from an on‐board imager: four‐dimensional cone‐beam CT and tumor tracking implications

**DOI:** 10.1120/jacmp.v12i2.3407

**Published:** 2011-02-01

**Authors:** Imad Ali, Nesreen Alsbou, Terence Herman, Salahuddin Ahmad

**Affiliations:** ^1^ Department of Radiation Oncology University of Oklahoma Health Sciences Center Oklahoma City OK 73104 USA; ^2^ Department of Electrical and Computer Engineering University of Oklahoma Norman OK 73019 USA

**Keywords:** projection, cone‐beam CT (CBCT), metal marker, motion tracks, motion correlation, image artifacts

## Abstract

The purpose of this work is to extract three‐dimensional (3D) motion trajectories of internal implanted and external skin‐attached markers from kV cone‐beam projections and reduce image artifact from patient motion in cone‐beam computed tomography (CBCT) from on‐board imager. Cone beam radiographic projections were acquired for a mobile phantom and liver patients with internal implanted and external skin‐attached markers. An algorithm was developed to automatically find the positions of the markers in the projections. It uses normalized cross‐correlation between a template image of a metal seed marker and the projections to find the marker position. From these positions and time‐tagged angular views, the marker 3D motion trajectory was obtained over a time interval of nearly one minute, which is the time required for scanning. This marker trajectory was used to remap the pixels of the projections to eliminate motion. Then, the motion‐corrected projections were used to reconstruct CBCT. An algorithm was developed to extract 3D motion trajectories of internal and external markers from cone‐beam projections using a kV monoscopic on‐board imager. This algorithm was tested and validated using a mobile phantom and patients with liver masses that had radio‐markers implanted in the tumor and attached to the skin. The extracted motion trajectories were used to investigate motion correlation between internal and external markers in liver patients. Image artifacts from respiratory motion were reduced in CBCT reconstructed from cone‐beam projections that were preprocessed to remove motion shifts obtained from marker tracking. With this method, motion‐related image artifacts such as blurring and spatial distortion were reduced, and contrast and position resolutions were improved significantly in CBCT reconstructed from motion‐corrected projections. Furthermore, correlated internal and external marker 3D‐motion tracks obtained from the kV projections might be useful for 4DCBCT, beam gating and tumor motion monitoring or tracking.

PACS numbers: 87.57.Q, 87.57.C‐

## I. INTRODUCTION

On‐board imaging (OBI)^(^ ^1^ ^,^ ^2^ ^)^ has proved clinical vitality for image‐guided radiation therapy. However, organ motion either involuntary such as respiration, gas motion in the rectum, and heart beating or voluntary patient relaxation produces image artifact in kV CBCT.^(3)^ Image quality degradation by motion in CBCT for OBI is greater than conventional CT^(^ ^4^ ^–^ ^8^ ^)^ because it takes a longer scanning time. The kV cone‐beam projections used in CBCT reconstruction are acquired over an extended scanning period of about 1 minute as required to perform a full rotation of the linear accelerator gantry.^(9)^ This period includes about 10 to 20 respiratory cycles in a regularly free‐breathing patient. Conventional CT images are acquired in short time snapshots producing small motion artifacts in the individual slices.^(10)^ Motion‐related image artifacts produce blurring, spatial distortion, poor contrast and position resolutions in CBCT, and limit its clinical value as a tool for tumor and soft tissue localization and visualization.^(4)^

Stereotactic body radiation therapy with a large single dose, or hypo‐fractionated doses, and intensity‐modulated radiation therapy techniques represent a challenge because of the need for more accurate patient setup and tumor localization. The delivery of large conformal doses may be limited by organ motion. For example, the treatment margins needed to correct for respiratory motion may require large planning target volumes (PTV) that includes normal tissue and critical structures. Several papers have reported about the employment of 4DCT^(^ ^11^ ^–^ ^13^ ^)^ that includes tumor motion in addition to the volumetric CT anatomical patient data to define PTV for treatment planning. One approach to minimize motion artifacts in CT is fast scanning using shorter scanner rotation time and multislice technology.^(^ ^10^ ^,^ ^14^ ^)^ Several other techniques were advocated to reduce motion artifacts by retrospectively correcting motion in the projections prior to CT reconstruction. Vedam et al.^(15)^ sorted retrospectively spiral CT‐images using external respiratory signal to reduce motion artifacts. Achenbach et al.^(16)^ used electrocardiogram‐gated spiral CT to improve contrast‐enhanced visualization of the coronary artery by reducing cardiac motion artifacts. Dhanantwari et al.^(5)^ used adaptive interference cancellation to remove motion artifacts in CT images. Ritchie et al.^(17)^ employed a pixel‐specific back‐projection to reduce doubling and streaking artifacts, and Lu et al.^(^ ^18^ ^,^ ^19^ ^)^ corrected motion in sinogram space prior to CT reconstruction. Some of above techniques has been applied successfully to improve image quality in conventional CT. However, the application of these techniques to CBCT from kV OBI systems is limited where the degradation of image quality by motion is stronger in the later one.^(20)^

There are several techniques to track tumor motion that include: (a) direct tumor tracking using radiographic or fluoroscopic imaging of internal fiducial markers implanted inside the patient,^(^ ^21^ ^–^ ^23^ ^)^ and (b) indirect tumor tracking by external marker^(24)^ or surface imaging,^(^ ^25^ ^,^ ^26^ ^)^ or respiratory sensor monitoring.^(27)^ One of the most commonly used systems for beam gating and 4DCT in radiotherapy is the Real Time Position System (RPM) provided by Varian Medical Systems (Varian Medical Systems, Palo Alto, CA) that is integrated on CT simulation and dose delivery machines. This gating system uses infrared imaging of an external marker attached to patient skin. However, the RPM system is limited by the imaging of an external marker where its motion is measured only in one‐dimension (anterior–posterior direction). Further, several studies have investigated correlation between external and internal marker motion and reported that though internal tumor motion might be correlated to external marker motion on the patient skin, motion amplitudes and phases could be different.^(^ ^24^ ^,^ ^27^ ^–^ ^31^ ^)^

In this work, an algorithm was developed to extract 3D motion trajectories of metal markers by tracking the position of seed markers in kV cone‐beam radiographic projections. This algorithm calculates six degrees of freedom required to determine both magnitude and direction of each marker using the monoscopic kV on‐board imager. The motion tracks from the projections were used to investigate correlation between external and internal marker motions in liver patients. Further, the trajectories of the markers were used to reduce motion from the projections, which were then used to reconstruct CBCT.

## II. MATERIALS AND METHODS

### A. kV on‐board‐imaging cone‐beam CT system

A kV OBI system mounted on a Varian Trilogy machine (Varian Medical Systems, Palo Alto, CA) was used to acquire cone‐beam projections. The OBI consists of a diagnostic quality kV X‐ray source and an amorphous‐silicon flat‐panel imager (PaxScan 4030CB, Varian Medical Systems) held by robotic arms mounted on the linac gantry. The source and imager are extended using the robotic arms and are set opposite to each other at 100 cm and 50 cm from isocenter, respectively, during image acquisition. The system rotates nearly 360° with the OBI at right angle with respect to the MV beam and takes nearly one minute to acquire about 650 projections. Two scanning modes are used: (a) full‐fan (FF) with a maximum field of view (FOV) of 17 cm thickness and 25 cm diameter, and (b) half‐fan (HF) with a FOV as large as 15 cm thickness and 50 cm diameter. The flat‐panel imager provides an effective imaging area of nearly $40\times 30\;{\text{cm}}^{2}$. Cone‐beam projections are usually acquired with a matrix of 1024×768 pixels, 16 bit‐depth and 7–10 frames per second. The kV, mA, and ms depend on the scanning protocol; for example, the HF and bowtie mode uses 125 kV, 80 mA and 25 ms. Bowtie filters (aluminum $2.8\;{g/\text{cm}}^{3}$) are used in CBCT both to reduce patient dose as well as to improve image quality by attenuating low‐energy scattered radiation.

### B. CAT phantom and moving platform

We used a commercially available phantom (Catphan 500, Phantom Laboratory, Salem, NY),^(32)^ mounted on a moving platform, to measure and quantify the motion of metal markers. Seed markers (1 mm diameter and 2 mm length) were attached to the Catphan which were visible with high contrast in the radiographic projections. The Catphan 500 is a cylinder with a diameter and length of 20 cm each. It contains several modules to test image quality parameters including CT number uniformity and linearity, contrast and spatial resolutions. These modules were used to evaluate image quality parameters of CBCT reconstructed before and after motion correction. The moving platform consists of a flat polystyrene surface that was attached to an arm of a driving motor. The motion amplitude and frequency of the motor arm were adjustable. In our measurement, the motion cycle of the moving platform was set at 15 cycles/min and 1.75 cm displacement amplitude in order to mimic actual patient respiratory motion.

### C. Metal marker tracking

The motion of internal and external markers was measured by tracking the marker positions in kV cone‐beam projections. The marker tracking is based on a normalized cross‐correlation image registration algorithm.^(33)^ This algorithm compares the intensity of a template image, *T*(*x, y*), of either an internal or an external metal marker with an image, *I*(*x, y*), from the sequence of radiographic projections acquired in a CBCT scan. The template image is obtained by selecting a region‐of‐interest (ROI) from one of the radiographic projections that includes the metal marker. This cross‐correlation algorithm registers the metal marker to similar objects in the projections by optimizing both intensity and shape in the ROI that includes the marker, and then determines the position of the point of maximum overlap. The position of maximum correlation is considered to represent the position of the metal marker in a projection. The error in localization of a marker position is about ± 0.5 pixel, which corresponds to about ± 0.13 mm at isocenter.

### D. Extraction of marker three‐dimensional motion

**Figure 1:**
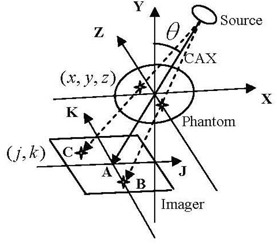
Schematic of the cone‐beam source, imager and patient coordinate (x,y,z) and imaging coordinates (j,k) systems.

**Figure 2:**
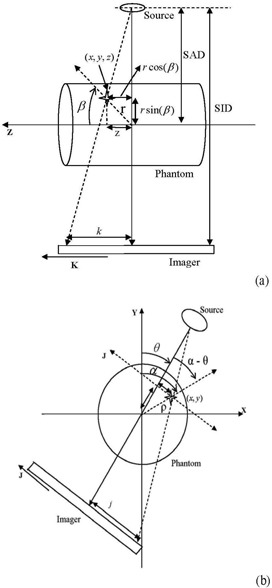
Geometric relationships ((a) and (b)) between patient (x,y,z) and imager (j,k).

The radiographic projections at different angular views, θ, are considered as snapshots in time. About 650 projections are acquired over 360° during a CBCT scan with nearly two projections per gantry angle for half‐fan scans. The projections are collected over a time period of about 1 minute, which is the time required for one full gantry rotation. In the imaging process using the on‐board CBCT system, each patient voxel (x,y,z) is projected onto a pixel (j,k) on a radiographic projection at a particular angular view, θ, as shown in Fig. 1. The isocenter point in a patient is considered as a patient voxel with (x=0,y=0,z=0) and the corresponding central pixel on the imager is (j=0,k=0). The patient is assumed to be composed of discrete voxels that have sides with equal distances (0.26 mm at isocenter). The marker position in the K‐direction represents patient's superior‐inferior (Z), and the J‐direction represents patient position in a plane perpendicular to the superior‐inferior direction (X‐Y), as shown in (Figs. 2a‐b). Using similarity of triangles in (Fig 2a), the relationship between ρ and j can be extracted and is given by the following equation:

(1) $$\frac{SAD-\rho \cos(\alpha -\theta )}{SID}=\frac{\rho \sin(\alpha -\theta )}{j}$$

where $\rho =r\sin(\beta )=\sqrt{x^{2}+y^{2}}$ is the radial distance in X‐Y plane $r=\sqrt{x^{2}+y^{2}+z^{2},\theta }$ is the projection view angle, $\alpha =\tan^{-1}(\frac{x}{y})$ is the angle which depends on the location of the patient voxel projection in X‐Y plane, $\beta =\tan^{-1}(\frac{\sqrt{x^{2}+y^{2}}}{z})$ is the angle between r and Z‐direction as shown in (Figs. 2a‐b), SAD is the distance from the source to the isocenter (100 cm), and SID is the distance between source and imager (150 cm).

From Eq. (1), the radial distance in the X‐Y plane is given by the following:

(2) $$\rho =c\frac{1}{\frac{\sin(\alpha -\theta )}{j}+\frac{\cos(\alpha -\theta )}{SID}}$$

where $c=\frac{\text{SAD}}{\text{SID}}$, which represents a scaling factor. Using similarity of triangles in Fig. 2(b), the relationship between the displacements z and k (in centimeters) of a patient voxel (x,y,z) and the corresponding image pixel (j,k) is obtained as follows:

(3) $$\frac{SAD-r\sin(\beta )\cos(\theta )}{SID}=\frac{z}{k}$$

From Eqs. (2) and (3), the relationship between the patient stationary voxels (x,y,z) and the imager pixels (j,k) is given by the following:

(4) $$\begin{array}{lll} x & = & \rho \sin(\alpha )=c\frac{\sin(\alpha )}{\frac{\sin(\alpha -\theta )}{j}+\frac{\cos(\alpha -\theta )}{SID}}\\ y & = & \rho \cos(\alpha )=c\frac{\cos(\alpha )}{\frac{\sin(\alpha -\theta )}{j}+\frac{\cos(\alpha -\theta )}{SID}}\\ z & = & c\;k(1-\frac{\rho \cos(\theta )}{SAD}) \end{array}$$

**Figure 3:**
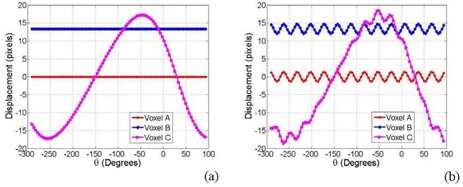
Sinograms (a) of three stationary voxels A, B and C (shown in Fig. 1); sinograms (b) of the voxels in (a) with a simple sinusoidal motion.

If we assume that the patient is not moving and thus his voxels are stationary, the position of the pixels on cone‐beam projections (j,k) changes from one view to another depending on the position of the corresponding voxels relative to the imaging isocenter and the projection view angle. A voxel in the patient that matches with the isocenter will be projected at the center of the imager (voxel A in Fig. 1) in all projections over 360° angular views, θ, with (j=0,k=0) according to Eq. (1). A patient voxel that is located at a displacement, z, off isocenter in the superior‐inferior direction will show up on the same position, k, on all projections and its position is independent of the angular view (voxel B in Fig. 1). However, the position of a patient voxel with (x,y) off isocenter on the imager, j, depends on both the distance from the isocenter and the imaging angular view (voxel C in Fig. 1). The positions on the imager, (j,k), are scaled with the ratio, $c=\frac{\text{SAD}}{\text{SID}}$, as given by Eq. (2). The position tracks of a patient voxel (i,j) can be represented by a sinogram where the distance between the voxel and isocenter in a projection is plotted against the view angle. (Figure 3a) shows the sinograms from a simulation of the tracks of the three stationary voxels on the imager: (A) represents the position track of the isocenter, (B) represents the track of a voxel that is displaced by 20 cm from isocenter along Z‐direction, and (C) represents a voxel in X‐Y plane that is displaced by 20 cm from isocenter. The position of a voxel along the superior‐inferior (z) direction is represented by a constant displacement along the k‐direction in all projections and it is not affected by the view angle as shown in (Fig 3a). However, the displacements measured by the imager of a patient voxel C vary sinusoidally as a function of the view angle. Voxels far away from isocenter produce larger displacements on the imager than those closer to isocenter.

**Figure 4:**
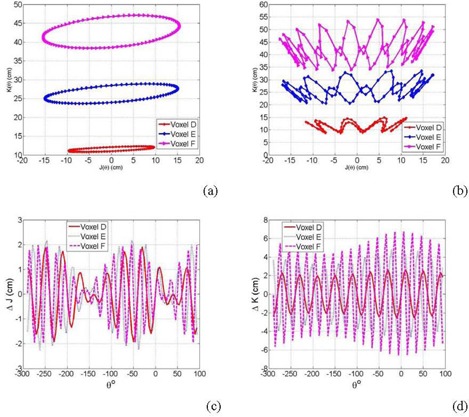
Two‐dimensional displacements (a) of three stationary voxels D, E and F; two‐dimensional displacements (b) of the voxels in (a) with a simple sinusoidal motion; displacements along J‐direction (c) of the three voxels D, E and F from a simple sinusoidal motion; displacements along K‐direction (d) from motion as in (c).

Patient motion causes offsets of the position of pixels on the imager that will superimpose on the stationary sinogram of the corresponding voxels. As shown in Fig. 3(b), the simulation demonstrates the sinograms of three moving voxels: A, B and C considering a cyclic‐simple sinusoidal respiration track with a displacement amplitude of 2 cm and a frequency of 12 Hz. The position of a stationary patient voxel from all projection creates a closed elliptical track as shown in Fig. 4(a), which illustrates a simulation of the J‐K positions for three stationary voxels D, E and F with radial distance (r), polar (α) and azimuthal (β) angles of (10, 40, 40), (20, 30, 30) and (30, 20, 20), respectively, on the imager. Figure 4 (b) shows the position of the three voxels D, E and F with simple cyclic motions with amplitudes of 2, 3 and 4 cm and frequencies of 9, 12 and 18 Hz, respectively. These motions complicate the position tracks that are obtained from the projections. However, the net displacements on the imager (Δj, Δk) resulting from the cyclic motions of the voxels D, E and F can be extracted by subtraction of displacements of the stationary $\left(j_{s},k_{s}\right)$ from the mobile $\left(j_{m},k_{m}\right)$ tracks, as shown by the simulations in (Figs. 4c‐d).

**Figure 5:**
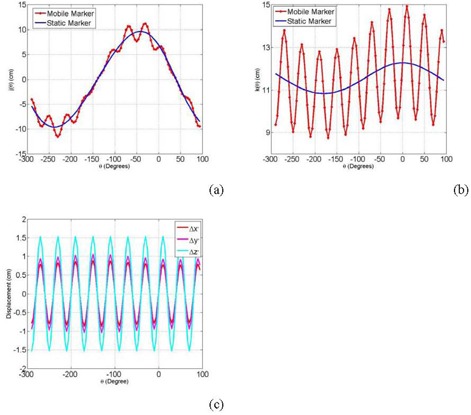
Filtering displacements of the stationary track using nonlinear curve fitting along J‐direction (a) and along K‐direction (b); the net motion components in X‐, Y‐ and Z‐directions (c).

In this algorithm, the marker track $\left(j_{m},k_{m}\right)$ including patient motion was extracted first from kV projections using normalized cross‐correlations, as explained previously. Then, the stationary track $\left(j_{s},k_{s}\right)$ was found with nonlinear curve fitting of a moving marker track $\left(j_{m},k_{m}\right)$ using the Levenberg‐Marquardt method,^(34)^ as shown in (Figs. 5a‐b). Then, (r, α, β) of the stationary marker were obtained from best‐fitting parameter of the measured mobile marker track with Eq. (5):

(5) $$\begin{array}{lll} J_{s}(r,\alpha ,\beta ) & = & \frac{r\sin(\beta )\sin(\alpha -\theta )}{c\;(1-r\sin(\beta )\cos(\alpha -\theta )/SAD)}\\ k_{s}(r,\alpha ,\beta ) & = & \frac{r\cos(\beta )}{c\;(r\cos(\beta )1-r\sin(\beta )\cos(\theta )/SAD)} \end{array}$$

The displacements (Δx', XXdy', Δz') due to motion in patient coordinate system can be calculated by subtracting the stationary $\left(x_{s},y_{s},z_{s}\right)$ from mobile $\left(x_{m},y_{m},z_{m}\right)$ voxel positions calculated from Eq. (4) according to the following:

(6) $$\begin{array}{lll} \Delta x^{\prime } & = & x(j_{m})-x(j_{s})\\ \Delta y^{\prime } & = & y(j_{m})-y(j_{s})\\ \Delta z^{\prime } & = & z(k_{m})-z(k_{s}) \end{array}$$

(Figure 5c) shows the net motion components in the different directions calculated by Eq. (6). The motion amplitudes, frequency and phase can be obtained by fitting the motion patterns from Eq. (6) with simple sinusoidal functions, as follow:

(7) $$\begin{array}{lll} \Delta x^{\prime } & = & A_{x}.\sin(2\pi f_{x}-\delta_{x})\\ \Delta y^{\prime } & = & A_{y}.\sin(2\pi f_{y}-\delta_{y})\\ \Delta z^{\prime } & = & A_{z}.\sin(2\pi f_{z}-\delta_{z}) \end{array}$$

where $A_{x},A_{y}$ and $A_{z}$ are motion amplitudes, $f_{x},f_{y}$ and $f_{z}$ are motion frequencies, and $\delta_{x},\delta_{y}$ and $\delta_{z}$ are phases in patient coordinate system in the X‐, Y‐, and Z‐directions, respectively.

### E. Correction of motion in CBCT

Position shifts (Δ j, Δ k) due to patient motion that are extracted from the sinogram of an internal marker were used as a transformation vector, (u, v), to map the position of the external marker at the various angular views for all projections from a CBCT scan. All pixels in each projection are shifted equally with the spatial shifts from the particular internal marker that is being tracked. Marker shifts along J‐axis of a projection removes marker motion in X‐Y plane using patient coordinate system. The marker shifts along K‐direction eliminates motion shifts in the superior‐inferior direction. The resultant 2D‐intensity map, $I_{\prime }(j_{s},k_{s},\theta )$, of the transformed radiographic projection at a certain angular view, θ, is given by the following equation:

(8) $$I^{\prime }(j_{s},k_{s},\theta )=I(j_{m}-u,k_{m}-v,\theta )$$

The transformed radiographic projections, I', as shown in Eq. (6), were used as input parameters to an image reconstruction program based on the back‐projection algorithm^(35)^ provided by the vendor (Varian Medical Systems). This technique of spatial mapping of the cone‐beam radiographic projections was applied to remove motion shifts from CBCT scans for both actual patient and Catphan phantom. The Catphan mounted on a moving platform was used to evaluate image quality of CBCT reconstructed from motion‐free projections. The marker motion track was used to map cone‐beam projections to eliminate motion shifts. Preprocessed projections were used for reconstruction of CBCT using Feldkamp back projection.^(35)^ Off‐line processing of the 650 projection images to extract marker motion track takes about 3 minutes using a MATLAB code (MathWorks, Inc., Natick, MA) that runs on a PC having an Intel Core Solo Processor U1400 of 1.2 GHz and 1 GB RAM. Preprocessing of the projection images to correct motion takes about 5 minutes. The reconstruction from the processed projections takes the same time as that from unprocessed projections.

### F. Liver patients

**Figure 6:**
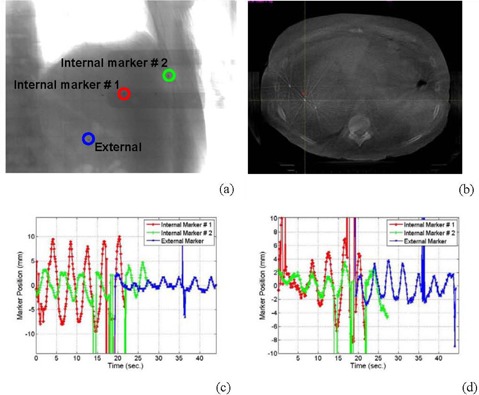
Internal and external seed markers in a radiographic projection (a) and a CBCT image (b); the internal (c) and external (d) markers’ motion shown in (a) and (b) parallel and perpendicular to the superior‐inferior, respectively.

In this work, we have investigated seed marker motion in four patients with liver masses. These patients had two to three seed markers that were implanted in the liver within and around the lesion area. At least one additional external marker was placed on patient skin anterior to the internal makers. These patients were treated with six fractions of 5 Gy per fraction using IMRT. (Figures 6a‐b) show a projection and a CBCT slice that include internal implanted and skin attached external seed markers used as surrogates for tumor localization and motion tracking, respectively. Marker motion was obtained by measuring the shifts in the marker position in cone‐beam radiographic projections acquired over 1 min, retrospectively. Depending on the location of the seed markers implanted in the patient, the extracted motion track of a marker may include about 7 to 12 respiratory cycles from the projections of one CBCT scan.

## III. RESULTS & DISCUSSION

### A. Motion extraction from cone‐beam projections

Figures 6 (c) and (d) show the motion tracks of two internal implanted seed makers and an external metal marker attached to the skin of a liver patient. Position shifts in Fig. 6(c) represent internal and external markers motion along z (superior‐inferior) direction. The curves in Fig. 6(d) represent marker motion in X‐Y plane. These 3D motion tracks were extracted from the projections acquired from one CBCT scan using half‐fan protocol. According to (Figs. 6c) and (d), patients have about 10 to 15 respiratory cycles in one minute from a regular breathing pattern. The number of respiratory cycles obtained from cone‐beam projections depends on the position of the marker relative to the OBI isocenter and scanning mode used to acquire CBCT. In half‐fan scans, the markers showed in the projections only when the patient side that includes the marker was imaged. For example, about five respiratory cycles were obtained for the internal maker #1, and seven cycles for the internal marker #2, as shown in (Figs 6c‐d). The markers were located at the right side of the patient and thus showed up nearly half the time in one scan. The external marker track also included seven respiratory cycles and it was located at the left side of the patient. This problem can be solved using full‐fan scans (< 25cm diameter), where the region of interest including seed markers shows up in each projection.

### B. Motion correlation between internal and external marker

**Figure 7:**
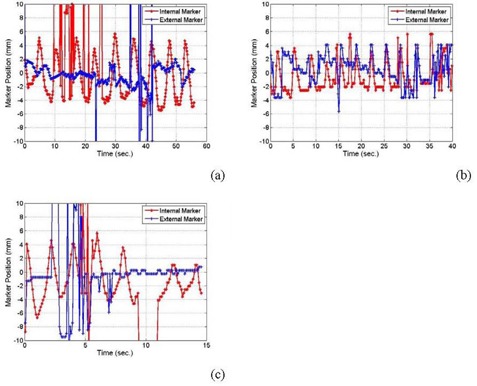
Patient motion in the superior‐inferior direction for patients # 2 (a), 3 (b), and 4 (c).

**Figure 8:**
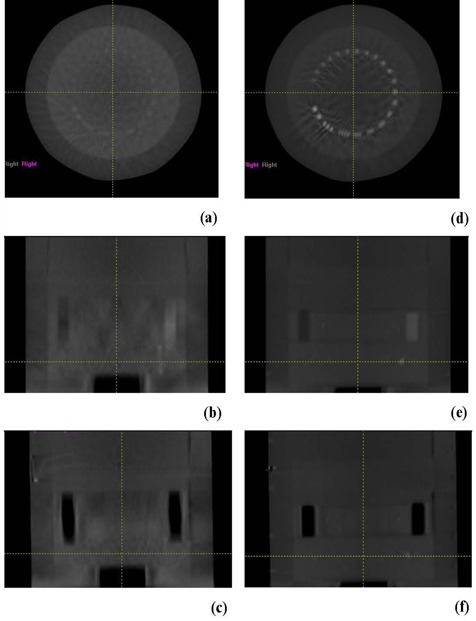
CBCT transverse, coronal and sagittal images, respectively, of the position resolution module of the Catphan reconstructed from cone‐beam projections: before motion correction (a‐c); after motion correction (d‐f).

The external and internal marker motion tracks for patient (1) (as shown in (Figs. 6c) and (d)) had the same frequency of respiration. However, internal markers had different motion amplitudes. For example, marker #1 close to the patient chest wall had an amplitude of nearly 3 mm, while marker #2 close to the diaphragm moved with an amplitude of about 9 mm. The external marker attached to patient skin had smaller amplitude (1.5 mm) than the internal markers, and also its motion track was out of phase with that of internal markers. The internal and external marker motion of patients 2 and 4 (Fig. 7(a) and (c)) were even not correlated. For two out of four patients, we found that external and internal marker motions correlated with each other. However, the motion amplitudes of the external markers were always smaller than that of the internal markers.

(Figures 6c) and (d) show outlier points on the measured data curve of motion shifts around 36 seconds. These points and others in Figs. 6 and 8 appeared where the tracking algorithm fails to detect the shadow of the metal marker in the corresponding radiographic projections. This failure of the algorithm to detect the metal marker was due to the existence of shadows that may have similar intensity‐gradient features as the marker in these particular radiographic projections. This problem was resolved using polynomial interpolation to predict patient motion in angular views in which the seed markers do not show up in the projections. The interpolated motion track predicted well the cyclic respiratory motion. However, patient relaxation or sudden motion can not be reproduced using this approach because it may not follow a particular cyclic pattern similar to patient respiration track that can be predicted by a fitting multiple polynomial function.

Similar to the results of numerous previous works,^(^ ^24^ ^,^ ^27^ ^–^ ^31^ ^)^ this study indicates that the motion of external marker may not correlate fully with internal marker motion. The motion correlation pattern varies from one patient to another and, even within the same patient, the motion amplitudes and phases vary between internal and external markers. For example, the motion tracks of patient 1 shown in (Figs. 6c‐d) demonstrate that the internal marker motion varies in amplitude as well as phase from the external marker. The motion amplitude of an internal marker close to the diaphragm is four times larger than the external marker in the superior‐inferior direction (see Fig. 6(c). The motion tracks obtained from cone‐beam projection in this work provide 3D motion components of internal and external markers, in contrast to the RPM system,^(^ ^15^ ^,^ ^36^ ^)^ which measures only a noncalibrated anterior‐posterior motion component of an infrared marker attached to the patient skin. In addition, tracking merely an external marker does not guarantee that its motion is correlated with internal tumor motion. The technique presented here can be useful to establish motion correlation between internal and external markers that can be used as a baseline for further patient motion monitoring or tracking. One possible application scenario of this approach is to establish radiographically a correlation of the motion between internal and external makers using cone‐beam projections considering differences in motion amplitudes and phases. Then, the external marker can be tracked using nonradiographic methods such as infrared^(15)^ or surface^(26)^ imaging for tumor motion monitoring, tracking or beam gating. In arc therapy, the internal marker motion can be extracted directly from kV cone‐beam projections and used for real‐time tumor tracking by imaging during treatment.

### C. Motion correction in CBCT

**Figure 9:**
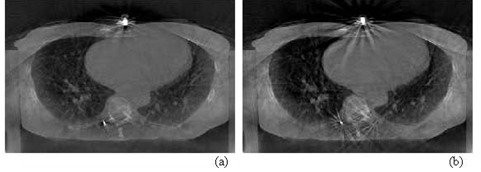
CBCT transversal images of a patient lung reconstructed from cone‐beam projections before (a) and after (b) motion correction.

Figures 8 and 9 show that CBCT reconstructed from projections after correction of patient motion has reduced artifacts than that reconstructed from projections without correction for motion. This technique reduced image artifacts associated with motion that include blurring, spatial distortion of objects, poor contrast and spatial resolution, as shown in CAT phantom. Lung border line and nodals were less blurred in CBCT images reconstructed from projections that were corrected for respiratory motion from tracking an implanted marker. In contrast to reconstruction of conventional CT from projections that are sorted in different motion phases using the motion track of an external marker,^(^ ^15^ ^,^ ^36^ ^)^ the approach used in this work uses internal marker motion to eliminate motion in the projections prior to the reconstruction of CBCT. CT images reconstructed from projections sorted at certain respiratory phases have residual motion because a respiratory phase does not represent a stationary state. The approach introduced in this work actually reduces motion artifacts in CBCT where projections are mapped onto a semi‐stationary position in which the motion amplitude is zero, instead of capturing the projections at different phases followed by reconstruction of CT at one phase or the other as it is done in phase‐sorted 4DCT. Another advantage of using projection mapping is that all projections from different angular views are used in the construction of motion‐corrected CBCT. However, 4DCBCT reconstructed from sorted projections include only the projection acquired in a certain respiration phase, which limits its image quality.

In the approach introduced in this work to correct respiratory motion, the whole patient body is assumed to move as a rigid body. Although the whole body may not be moving altogether rigidly, the ROI which includes the seed marker and the surrounding lesion will be moving similar to the marker within. Thus, anatomical mapping will be accurate for the ROI that includes the seed marker and tumor. This technique uses actual motion of a marker implanted in the ROI, while in CT reconstructed from phase‐sorted projections, an external marker is often used which could be out of phase with internal tumor motion. In contrast to other algorithms based on motion simulation or modeling^(^ ^23^ ^,^ ^37^ ^,^ ^38^ ^)^ to correct patient motion, this technique uses the measured specific‐patient motion from implanted seed marker. The track of only one seed marker was used here to correct for patient motion. Furthermore, with this projection mapping technique, an average shift from more than one seed marker can be used to correct patient motion in the projections, or the motion of several markers can be used to map the corresponding ROI's locally in the projections in order to reduce differential motion at various patient parts. These techniques may be interesting for a future investigation to test the ability to perform differential motion correction in the tumor and other regions of interest.

Several works have been reported about markerless motion tracking techniques which are based on flouroscopic imaging,^(^ ^39^ ^–^ ^42^ ^)^ radiographic projections,^(^ ^43^ ^–^ ^45^ ^)^ external skin surface imaging,^(^ ^25^ ^,^ ^26^ ^)^ or respiratory sensor monitoring.^(27)^ These approaches used an anatomical surrogate, surface features or air flow of the patient for tracking. Markerless tracking avoids drawbacks due to the risk of clinical complications associated with marker implantation, such as pneumothorax^(46)^ and marker migration.^(47)^ However, radiographic markerless tracking techniques are limited by insufficient motion correlation of the surrogate with tumor, and lack of contrast and border definition and shape variation of the surrogate in the various imaging views. In contrast, with tracking of anatomical surrogates specifically in cone‐beam projections,^(^ ^43^ ^–^ ^45^ ^)^ a metal marker with high‐contrast resolution and similar features in most angular views works well with automatic tracking algorithms, as we have demonstrated in this work. Furthermore, the motion of internal markers varies depending on the location of the markers within the liver (as shown in (Figs. 6c‐d)), where the motion amplitude of the markers implanted close to the diaphragm was larger than that of the markers implanted on the tumor side far away from the diaphragm. The motion amplitude and phase and variation in the motion of different regions in the tumor can be more accurately quantified by automated tracking of well‐defined small markers than anatomical surrogates.

This technique is limited by imaging blind spots of marker motion in a plane parallel to the CAX line. However, this motion component does not influence image quality and thus is not required to reconstruct motion‐free CBCT using the algorithm developed here. Further, the probability that one or two internal markers will have the motion in this plane is small considering the whole imaging solid angle. If a blind spot takes place in one angular view, motion signal will be recovered from other angular view and thus the marker motion track is reconstructed from the available data. Another limitation of the use of marker motion from cone‐beam projections is the visibility of the markers in all projections. This can be resolved by using full‐fan mode to image only appropriate ROI that includes the tumor and seed markers. Considering the 3D volumetric imaging and motion information by marker tracking from cone‐beam projections, our technique extends the use of the kV OBI to an additional dimension to perform 4DCBCT. Furthermore, the amplitudes of motion extracted in real time from internal markers just prior to dose delivery can be used to define accurate margins for the treatment planning target and perform adaptive radiation therapy.

In contrast with fluoroscopy or other radiographic techniques, using cone‐beam projections to obtain tumor motion requires no additional dose to the patient other than that used for CBCT imaging. Furthermore, sagging of the heavy linac gantry during the rotation around the patient in imaging produces shifts that add small displacements to the position of the voxels projected on the imager.^(48)^ The sagging shifts have to be subtracted from the sinogram of each seed marker in order to obtain net displacements due to respiratory motion.

## IV. CONCLUSIONS

In this work, an algorithm to extract 3D internal and external marker motion from kV cone‐beam radiographic projection was developed. Although internal and external marker motion might correlate with each other and have similar motion frequency, the markers were found to have different motion amplitudes and phases in liver patients. 3D‐motion of internal seed markers provides actual tumor position variation due to respiratory motion. Combining this information about tumor motion and 3DCBCT allows not only accurate patient setup for IGRT procedures, but also real‐time prediction of tumor motion trajectory just before starting patient treatment. The marker motion track from cone‐beam projections provides motion information that can be compared with the motion of other external markers such as the RPM infrared signal to test internal and external marker correlation and validation of beam gating or tumor tracking. Internal marker motion can be extracted directly from kV cone‐beam projections and used for online tumor tracking by imaging during treatment in arc therapy. Motion produces strong image artifacts in kV CBCT such as blurring, spatial distortion, poor contrast and position resolutions. The technique developed and tested in this work to track and correct marker motion in cone‐beam projections prior to reconstruction eliminates motion‐related image artifacts.
